# Histones released by NETosis enhance the infectivity of SARS-CoV-2 by bridging the spike protein subunit 2 and sialic acid on host cells

**DOI:** 10.1038/s41423-022-00845-6

**Published:** 2022-03-10

**Authors:** Weiqi Hong, Jingyun Yang, Jun Zou, Zhenfei Bi, Cai He, Hong Lei, Xuemei He, Xue Li, Aqu Alu, Wenyan Ren, Zeng Wang, Xiaohua Jiang, Kunhong Zhong, Guowen Jia, Yun Yang, Wenhai Yu, Qing Huang, Mengli Yang, Yanan Zhou, Yuan Zhao, Dexuan Kuang, Junbin Wang, Haixuan Wang, Siyuan Chen, Min Luo, Ziqi Zhang, Tianqi Lu, Li Chen, Haiying Que, Zhiyao He, Qiu Sun, Wei Wang, Guobo Shen, Guangwen Lu, Zhiwei Zhao, Li Yang, Jinliang Yang, Zhenling Wang, Jiong Li, Xiangrong Song, Lunzhi Dai, Chong Chen, Jia Geng, Maling Gou, Lu Chen, Haohao Dong, Yong Peng, Canhua Huang, Zhiyong Qian, Wei Cheng, Changfa Fan, Yuquan Wei, Zhaoming Su, Aiping Tong, Shuaiyao Lu, Xiaozhong Peng, Xiawei Wei

**Affiliations:** 1 Laboratory of Aging Research and Cancer Drug Targeting, State Key Laboratory of Biotherapy and Cancer Center, National Clinical Research Center for Geriatrics, West China Hospital, Sichuan University, No. 17, Block 3, Southern Renmin Road, Chengdu, Sichuan 610041 China; 2 National Kunming High-level Biosafety Primate Research Center, Institute of Medical Biology, Chinese Academy of Medical Sciences and Peking Union Medical College, Yunnan, China; 3 Westvac Biopharm Co., Ltd. No. 618, Fenghuang Road, Shuangliu District, Chengdu, Sichuan China; 4 Division of Animal Model Research, Institute for Laboratory Animal Resources, National Institutes for Food and Drug Control, 102629 Beijing, China; 5 State Key Laboratory of Medical Molecular Biology, Department of Molecular Biology and Biochemistry, Institute of Basic Medical Sciences, Medical Primate Research Center, Neuroscience Center, Chinese Academy of Medical Sciences, School of Basic Medicine Peking Union Medical College, Beijing, China

**Keywords:** COVID-19, SARS-CoV-2, neutrophil extracellular traps, histones, sialic acid, Immunology, Infection

## Abstract

Neutrophil extracellular traps (NETs) can capture and kill viruses, such as influenza viruses, human immunodeficiency virus (HIV), and respiratory syncytial virus (RSV), thus contributing to host defense. Contrary to our expectation, we show here that the histones released by NETosis enhance the infectivity of SARS-CoV-2, as found by using live SARS-CoV-2 and two pseudovirus systems as well as a mouse model. The histone H3 or H4 selectively binds to subunit 2 of the spike (S) protein, as shown by a biochemical binding assay, surface plasmon resonance and binding energy calculation as well as the construction of a mutant S protein by replacing four acidic amino acids. Sialic acid on the host cell surface is the key molecule to which histones bridge subunit 2 of the S protein. Moreover, histones enhance cell–cell fusion. Finally, treatment with an inhibitor of NETosis, histone H3 or H4, or sialic acid notably affected the levels of sgRNA copies and the number of apoptotic cells in a mouse model. These findings suggest that SARS-CoV-2 could hijack histones from neutrophil NETosis to promote its host cell attachment and entry process and may be important in exploring pathogenesis and possible strategies to develop new effective therapies for COVID-19.

## Introduction

Severe acute respiratory syndrome coronavirus 2 (SARS-CoV-2) causes an infectious viral respiratory disease known as COVID-19, severely affecting global public health year-round. SARS-CoV-2 is an enveloped virus with a single-stranded positive (+)-sense RNA genome of ∼30 kb. The four structural proteins of SARS-CoV-2 are the spike (S), membrane (M), envelope (E), and nucleocapsid (N) proteins [1]. The S protein of SARS-CoV-2 consists of two subunits, S1 and S2, which are responsible for receptor recognition and the cell membrane fusion process, respectively [2]. SARS-CoV-2 first binds to the ACE2 receptor on the host cell surface via the virion RBD in S1. Viral attachment and host protease cleavage facilitate conformational changes in the S protein and the insertion of the fusion peptide of the S protein into the target membrane. Subsequently, the heptad repeat 1 (HR1) and 2 (HR2) domains in S2 interact with each other to form a stable six-helix bundle fusion core and eventually accelerate the viral and cellular membrane fusion process [3]. Research on the high neutralization potency of human monoclonal antibodies that bind to the SARS-CoV-2 S protein but do not bind RBD suggested the existence of other (co)receptors or mechanisms for the entry of SARS-CoV-2 into cells [4]. Recent studies revealed that S1 of SARS-CoV-2 could also bind to other receptors or coreceptors, such as neuropilin-1, heparan sulfate, HDL-scavenger receptor B type 1 (SR-B1), tyrosine-protein kinase receptor UFO (AXL) and CD147, to facilitate cell entry and potentiate infectivity [5–10]. However, no reports indicate that S2 of SARS-CoV-2 can interact with host factors.

Neutrophils release neutrophil extracellular traps (NETs) in response to exogenous invading bacteria, viruses, or other pathogens. NETs can capture and kill viruses, such as influenza viruses, HIV and RSV, thus contributing to host defense [11–13]. Many viruses stimulate neutrophils to directly produce NETs. For example, neutrophils detect HIV nucleic acids via Toll-like receptor (TLR) 7 and TLR 8 [12] and recognize the fusion protein of RSV via TLR 4 [14], subsequently releasing NETs. The net-like chromatin backbone of NETs can bind and immobilize viruses partly by electrostatic attraction [12]. In addition, antimicrobial molecules in NETs, such as myeloperoxidase (MPO) and α-defensin, have antiviral activity that can inactivate viruses [15]. As the most abundant proteins in NETs, extracellular histones comprise five core subunits (linker histone H1, H2A, H2B, H3, H4), contain positively charged amino acids, and exert antiviral effects [16, 17]. The citrullination of histones is considered to be a specific marker of NETosis [18]. However, some studies reported elevated histones with a positive charge in NETs instead of citrullinated histones [19–22]. Components of NETs can also stimulate and activate other immune cells, such as plasmacytoid dendritic cells and T lymphocytes, to execute antiviral effector mechanisms [23, 24]. However, in addition to the positive effect on innate defense, unbalanced NET formation causes tissue damage through adverse proinflammatory effects. For example, the positively charged histones of NETs are directly cytotoxic to epithelial and endothelial cells and contribute to thrombosis [25–27], and the cytotoxicity caused by histones or NETs is reversed by highly negatively charged PSA [25]. Recent studies reported increased levels of NETs and extracellular histones in the blood, tracheal aspirate, and lung tissues of patients with severe COVID-19 [26, 28]. NETs were found in the airway compartment and neutrophil-rich areas of the interstitium, while NET-prone primed neutrophils were found in arteriolar microthrombi in the lung tissue of COVID-19 patients [29]. Moreover, SARS-CoV-2 can directly trigger the spontaneous release of NETs [30]. However, it is still unclear whether NETs or neutrophil NETosis impact the infectivity of SARS-CoV-2.

Here, we show that histones released from neutrophil NETosis enhance the infectivity of SARS-CoV-2. The histone H3 and H4 selectively bind to S2 of SARS-CoV-2 with four acidic amino acids. Moreover, we emphasize that sialic acid on host cells is a key molecule to which histone forms a bridge from S2 promotes the membrane fusion process, thereby enhancing SARS-CoV-2 infectivity. Finally, the effect of histones on the infectivity of SARS-CoV-2 was also observed in a SARS-CoV-2-induced acute respiratory distress syndrome mouse model.

## Results

### Histones released by neutrophils enhance the infectivity of SARS-CoV-2

**Fig. 1:**
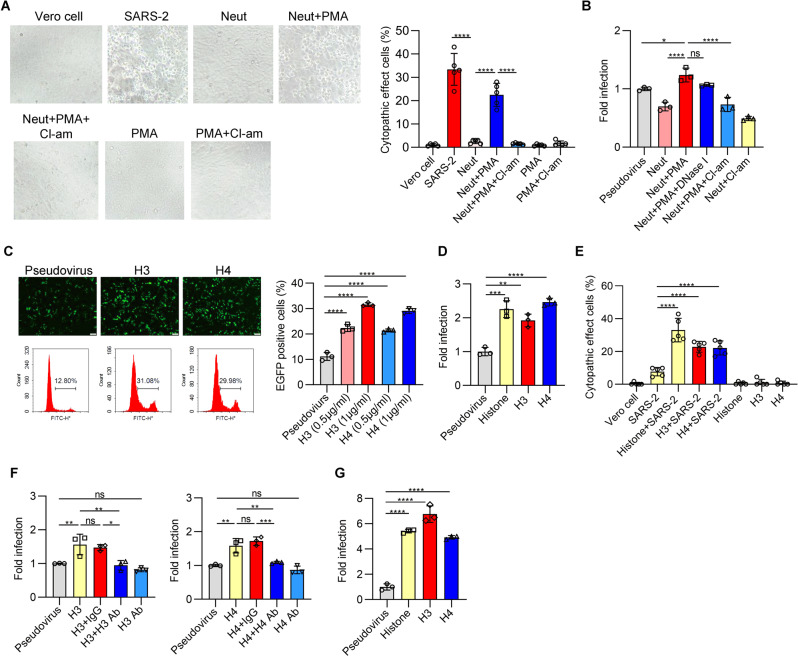
Histones released by neutrophils enhance SARS-CoV-2 infectivity. **A** The supernatant of PMA-activated neutrophils enhanced SARS-CoV-2 infectivity. Human neutrophils (1 × 10^5^ cells) were preincubated with SARS-CoV-2 (MOI = 0.05) in the presence of PMA or PMA plus Cl-amidine or untreated at 37 °C for 3 h, and then the supernatant was collected. Additionally, the supernatants were prepared and collected with SARS-CoV-2 alone in the absence of neutrophils, or with PMA alone or PMA and Cl-amidine as controls. Then, the supernatant was added to Vero E6 cells as described in the Methods. After 48 h, the cytopathogenic effects (CPEs) were recorded using a microscope, and the percentages of cells with cytopathogenic effects were calculated accordingly. Vero cell: Vero cells alone without SARS-CoV-2; SARS-2: infection with SARS-CoV-2 alone; Neut: Vero cells infected with the supernatant of neutrophils preincubated with SARS-CoV-2 alone; Neut+PMA: Vero cells infected with the supernatant of neutrophils preincubated with SARS-CoV-2 in the presence of PMA; Neut+PMA + Cl-am: Vero cells preincubated with SARS-CoV-2 in the presence of PMA + Cl-am; PMA: Vero cells alone without SARS-CoV-2 in the presence of PMA; PMA + Cl-am: Vero cells alone without SARS-CoV-2 in the presence of PMA + Cl-am. **B** The infectivity of luciferase-expressing pseudovirus was enhanced by the supernatant from PMA-activated neutrophils and others as described in (**A**). The data were normalized to the respective infectivity of luciferase-expressing pseudovirus incubated with pseudovirus alone in ACE2-expressing HEK-293T (293T/ACE2) cells. **C** 293T/ACE2 cells were infected with EGFP-expressing pseudovirus preincubated with histone H3 (0.5 and 1 μg/ml) or H4 (0.5 and 1 μg/ml), and then the mixture was incubated with 293T/ACE2 cells for 48 h to analyze infectivity as described in the Methods. The percentages of EGFP-positive cells were quantified by flow cytometry, and representative fluorescence photos and flow cytometry images are shown. **D** 293T/ACE2 cells were infected with luciferase-expressing pseudovirus preincubated with total histone (5 μg/ml), histone H3 (1 μg/ml) or H4 (1 μg/ml) as described in the Methods. **E** The percentages of CPE in Vero cells after SARS-CoV-2 (MOI = 0.01) was preincubated with total histone, histone H3, or H4 at 37 °C for 3 h, and then the mixture was incubated with Vero cells for 48 h to analyze the infectivity. **F** Histone H3 or H4 was pretreated with an antibody against histone H3 or H4. Then, the mixture was incubated with pseudovirus, and the effect of H3 and H4 on the infectivity of the pseudovirus was evaluated. The isotype was added as a control. **G** The infectivity of the pseudovirus preincubated with histone H3 or H4 was determined in human lung epithelial Calu-3 cells. Scale bars represent 100 μm in (**C**). In terms of statistical methods, one-way ANOVA followed by Tukey’s multiple comparison post hoc test was conducted through (**A**–**G**). All error bars represent the SEM. *P < 0.05; **P < 0.01; ***P < 0.001; ****P < 0.0001; ns not significant

To explore whether neutrophil NETosis could inhibit SARS-CoV-2 infectivity, we performed viral infection assays using live SARS-CoV-2 and Vero E6 cells. SARS-CoV-2 preincubated with neutrophils activated with phorbol myristate acetate (PMA, a potent inducer of NETosis) or unactivated neutrophils was collected to infect Vero cells. The percentages of cells with cytopathogenic changes were calculated as reported in our previous study [31]. Contrary to our expectation, PMA-activated neutrophils failed to block SARS-CoV-2 infectivity compared with the unactivated neutrophil group (Fig. 1A). Preincubation of SARS-CoV-2 with unactivated neutrophils alone significantly decreased the percentages of infected Vero cells (Fig. 1A). In addition, the enhanced infectivity of SARS-CoV-2 induced by PMA-activated neutrophils was eliminated by treatment with a NETosis inhibitor (Cl-amidine, an inhibitor of PAD4). Treatment with PMA or Cl-amidine alone did not cause cytopathogenic effects (Fig. 1A). These findings indicate that the increased infectivity of SARS-CoV-2 may be related to neutrophil NETosis.

In the next set of experiments, we established a pseudovirus system encoding the SARS-CoV-2 spike protein with a luciferase reporter or EGFP reporter gene to further confirm whether neutrophil NETosis enhances SARS-CoV-2 infectivity. Accordingly, pseudovirus pretreated with PMA-activated neutrophils, compared with unactivated neutrophil-treated pseudovirus or pseudovirus alone, induced increased infectivity in ACE2-expressing HEK-293T cells (Fig. 1B). Moreover, the enhanced infectivity of the pseudovirus induced by PMA-activated neutrophils was also inhibited by treatment with Cl-amidine but not by DNase I treatment (Fig. 1B), indicating that DNA components in NETosis might be less relevant in enhancing the infectivity of the pseudovirus.

Histones are also the major components that form NETs and consist mainly of positively charged amino acids; thus, we hypothesized that positively charged histones might attach to the anionic binding sites of the viral spike protein, subsequently acting as a key mediator to enhance the cell attachment and cell entry of the virus. Interestingly, we found that the pseudovirus pretreated with total histone, histone H3 or H4 at a lower concentration exhibited increased infection percentages compared with that of the control group in the pseudovirus system encoding the SARS-CoV-2 spike protein with an EGFP reporter gene (Fig. 1C) or luciferase reporter (Fig. 1D). Histone H2A or H2B is less effective in enhancing the infectivity of the pseudovirus; however, they were also workable at a rather high concentration (Fig. S1). Therefore, in this study, we focused on the potential role of histone H3 or H4 in enhancing SARS-CoV-2 infectivity.

Additionally, we found that total histone, histone H3 or histone H4 were able to enhance the infectivity of live SARS-CoV-2 in Vero cells, whereas histones alone at the same concentration did not cause cell death (Fig. 1E). Utilizing a blocking antibody against extracellular histones is also an approach to investigate histone-mediated activity [25]. We found that the infectivity of pseudovirus mediated by histone H3 or H4 was significantly inhibited after the pretreatment of histones with antibodies against histone H3 or H4 (Fig. 1F and Fig. S2). In addition, total histone, histone H4 or histone H3 enhanced the infectivity of the pseudovirus in human lung epithelial cells (Fig. 1G). In addition, the enhanced infectivity mediated by histones was not found in the cells without ACE2 expression, indicating that the phenomenon may be ACE2-dependent (Fig. S3).

### Histones H3 and H4 selectively interact with SARS-CoV-2 S2

**Fig. 2:**
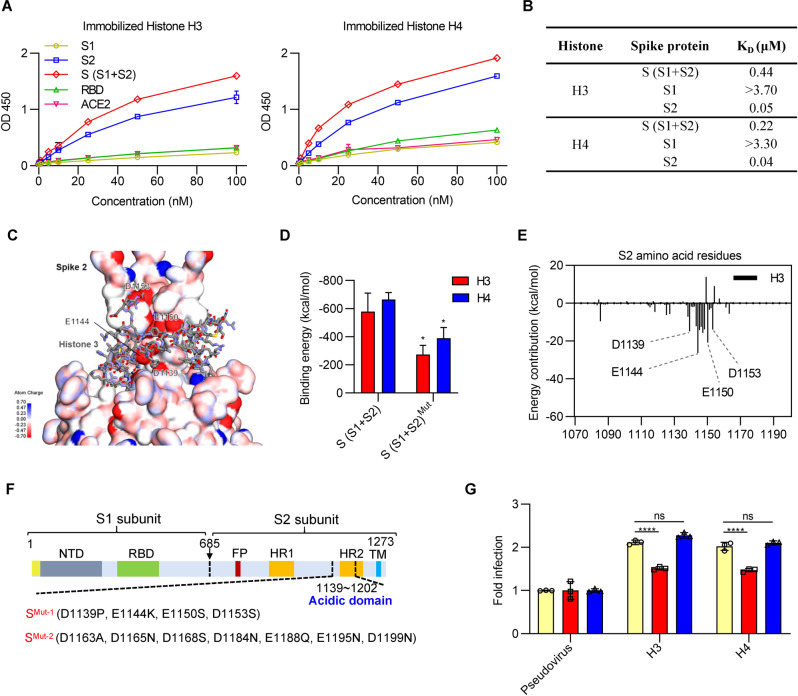
Histone H3 and H4 selectively interact with the SARS-CoV-2 S2 protein. **A** The bindings of spike, subunit 1, subunit 2, RBD or ACE2 proteins to histone H3 or histone H4 were performed by the binding assay, as described in Methods. The absorbance was measured at 450 nm. **B** Summary of the dissociation constant (*K*_D_) of the real-time binding profile between spike protein (S1 + S2), S1, S2, and histone H3 or H4 (Biacore). **C** Electrostatic surface rendering of the C-terminus of subunit 2 protein (PDB ID 6VSB) in complex with histone H3 (shown in sticks). Blue and red surfaces indicate electropositive and electronegative surfaces, respectively. The important binding residues are labeled. **D** Average interaction binding energies between histone H3 or H4 and SARS-CoV-2 S protein and their mutants (D1139P, E1144K, E1150S, and D1153S). **E** Calculated energy contributions of each amino acid residue of S2 that can interact with histone H3. **F** The construction of the mutations in the acidic C-terminal domain of S2. Mut-1, which covered the negatively charged amino acids, included mutations at D1139P, E1144k, E1150S, and D1153S. Mut-2, which covered the other negatively charged amino acids, included D1163A, D1165N, D1168S, D1184N, E1188Q, E1195N, and D1199N. **G** Quantitative analysis of the infectivity of pseudovirus S^Mut-1^ (red) or pseudovirus S^Mut-2^ (blue) preincubated with histone H3 or H4. The pseudovirus without mutation (yellow) was used as a control. Student’s *t* test was performed for (**D**), and two-way ANOVA followed by Tukey’s multiple comparisons test was conducted for (**G**). All error bars represent the SEM. *P < 0.05; **P < 0.01; ***P < 0.001; ****P < 0.0001; ns not significant

It is conceivable that the increased infectivity mediated by the extracellular histones may result from their bridging between the spike protein of SARS-CoV-2 and certain binding sites on the host cell surface. To characterize the potential binding sites on the spike protein of the virus with histones, including histones H3 and H4, we first calculated the isoelectric point (pI) of the full-length S protein (aa, 16-1213) and its S1/S2 subunits (aa 16–685) (aa 686–1213) by using a tool from Expasy (https://web.expasy.org/compute_pi). We found that the isoelectric points (pIs) of the full-length S protein, S1 and S2 were 6.30, 8.27, and 5.23, respectively. Furthermore, several experiments were performed to investigate the interaction between the spike protein of SARS-CoV-2 and histones. We found that S2 or the ectodomain (S1 and S2) of the spike protein exhibited much stronger binding to immobilized histone H3 or H4 in a dose-dependent manner than S1, RBD or ACE2 protein (Fig. 2A). The binding of histone H3 or H4 with the subunit 2 protein or ectodomain (subunit 1 and 2 protein) was further confirmed using surface plasmon resonance (Biacore) (Fig. 2B and Fig. S4). These findings suggest that histone H3 or H4 may selectively bind to S2.

We next carried out molecular simulation to investigate the interaction between histones and the SARS-CoV-2 spike (S) protein. We performed rigid-body protein–protein docking using the ZDOCK algorithm and selected the optimal pose from the largest cluster with a high ZRANK score. The C-terminus of the SARS-CoV-2 S2 protein lies in a group of negatively charged amino acid residues that represent a putative binding site that could interact with histones (Fig. 2C and Fig. S5A). We calculated an electrostatic potential map of the C-terminus of the S protein (from PDB ID 6VSB), which revealed an extended electronegative surface consistent with the preferred histone-binding site (Fig. 2C and Fig. S5A, B). Notably, the putative histone-binding surface is distant from the ACE2-binding site and is not obstructed in either the active or inactive conformation. The calculated binding energy of H3 and H4 with the SARS-CoV-2 S protein was −579.6 kcal/mol and −665.3 kcal/mol (Fig. 2D) and was reduced by acidic amino acid substitution (see Fig. 2F below). Evaluation of S protein-histone contacts and energy contributions suggests strong interactions with the negatively charged amino acids D1139, E1144, E1150, and D1153 (Fig. 2E and Fig. S5C). Other amino acids, notably S1147 and F1148, can coordinate histones through hydrogen and hydrophobic interactions. Moreover, mutagenesis was performed by amino acid substitution of the acidic C-terminal domain of subunit 2 protein (Fig. 2F). The pseudovirus with Mut-1 (covering the negatively charged amino acids, including D1139P, E1144k, E1150S, and D1153S) showed a reduction in the enhancement of infectivity by the addition of histone H3 or H4 (Fig. 2G). Furthermore, Mut-2 (covering the other negatively charged amino acids, including D1163A, D1165N, D1168S, D1184N, E1188Q, E1195N, and D1199N) had no effect on the infectivity of the virus alone or in the presence of histones (Fig. 2G). These findings demonstrated that the negatively charged amino acids (D1139P, E1144k, E1150S, and D1153S) in the acidic C-terminal domain may play an important role in the enhanced virus infectivity mediated by histones.

### Histone H3 and H4 bridge S2 and sialic acid on host cells to promote membrane fusion

**Fig. 3:**
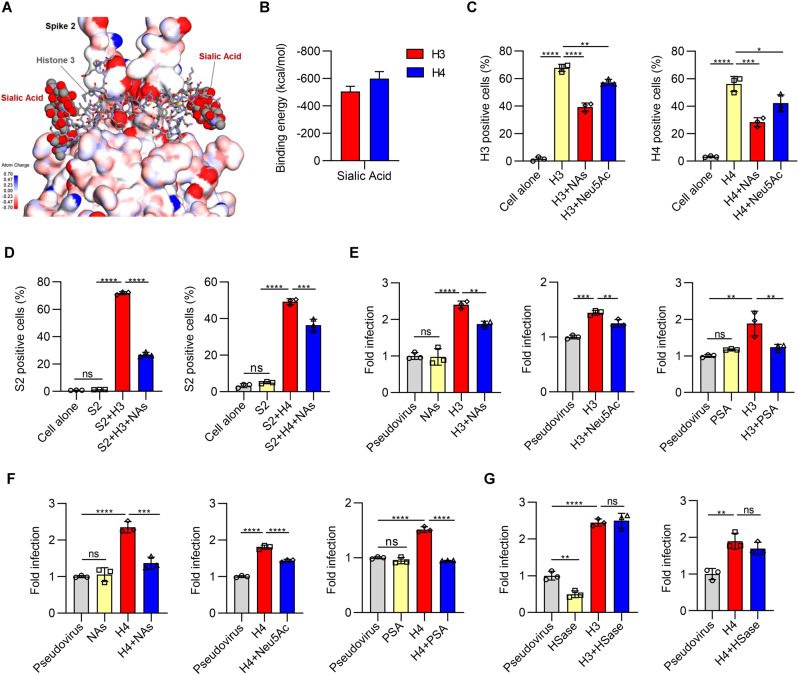
Histone H3 and H4 bridge S2 and sialic acid on host cells and promote infectivity. **A** A molecular model of histone H3 forming multivalent interactions with both S2 and sialic acid. Sialic acids are represented using a standard sphere model. **B** Average interaction binding energies between sialic acid and histones H3 and H4. **C** Flow cytometry analysis of the percentages of biotin-tagged histone H3 or H4 binding to Calu-3 cells pretreated with NAs or in the presence or absence of Neu5Ac. NAs: neuraminidase; Neu5Ac: sialic acid. **D** The biotin-tagged S2 proteins were preincubated with histone H3 or H4 and then added to Calu-3 cells pretreated with NAs. The percentages of biotin-positive Calu-3 cells were determined by flow cytometry. (**E** and **F**) ACE2-positive HEK-293T cells pretreated with NAs (left) or in the presence of Neu5Ac (middle) or polysialic acid (right) were observed for the infectivity of pseudovirus preincubated with histone H3 (**E**) or H4 (**F**). PSA: polysialic acid. **G** The effects of heparin lyase (HSase) treatment of 293 T/ACE2 cells on the infection of pseudovirus preincubated with histone H3 (left) or H4 (right). One-way ANOVA followed by Tukey’s multiple comparison post hoc test was conducted through (**C**–**G**). All error bars represent the SEM. *P < 0.05; **P < 0.01; ***P < 0.001; ****P < 0.0001; ns not significant

In the next set of experiments, we identified the potential binding site on the host cell surface, which might act as a key mediator in bridging the SARS-CoV-2 S2 with the host cell surface via extracellular histones. Sialic acids are located mainly on the ends of glycans in glycoproteins or glycolipids on the cell surface, which is one of the important components maintaining the negative charge of the cell surface [32]. We carried out molecular dynamics simulations (100 ns long) of the interactions of histones with both S2 and sialic acid. Our results suggest that histone H3 or H4 could form multivalent interactions with both S2 and sialic acid (Fig. 3A and Fig. S6). Sialic acid interacts mainly with the positively charged residues at the ends of both histones H3 and H4. The calculated binding energy of histones H3 and H4 with sialic acid is −505.6 kcal/mol and −600.4 kcal/mol (Fig. 3B), respectively. Furthermore, to investigate the crucial binding of surface sialic acid with histones, we removed cell surface sialic acid with neuraminidase (NA) or pretreated histones with sialic acid to block their binding with cell surface sialic acid (Fig. 3C). Both the treatment of cells with NA and the pretreatment of histones with sialic acid significantly inhibited the cell attachment of histone H3 and H4 in human lung cells, as detected by flow cytometry (Fig. 3C). Additionally, similar results were found utilizing the subunit 2 protein-histone complex in the cell attachment assay. We mixed S2 with histone H4 or histone H3 to form the complex and then added it to human lung epithelial cells. The S2-histone (H3 and H4) complex was able to bind to the cell surface, and the binding was blocked by NA (Fig. 3D). Importantly, S2 could hardly bind to the host cell surface without the addition of histone H4 or histone H3 (Fig. 3D). Moreover, we found that the increase in infectivity of pseudovirus mediated by histone H3 or histone H4 was also blocked by NA, free sialic acid or polysialic acid (PSA) (Fig. 3E, F). However, we found that the treatment of cells with heparin lyases to remove heparin on the cell surface did not reduce the infectivity mediated by histone H3 or histone H4, although the treatment reduced the infectivity of the pseudovirus without histones (Fig. 3G). Our findings suggest that histones may bridge S2 and sialic acids on the host cell surface and consequently enhance the infectivity of SARS-CoV-2, which is a different system from the heparin/heparin sulfate-binding site adjacent to the ACE2-binding site of S1 that directly bonds to heparin sulfate on the cell surface [7].

**Fig. 4:**
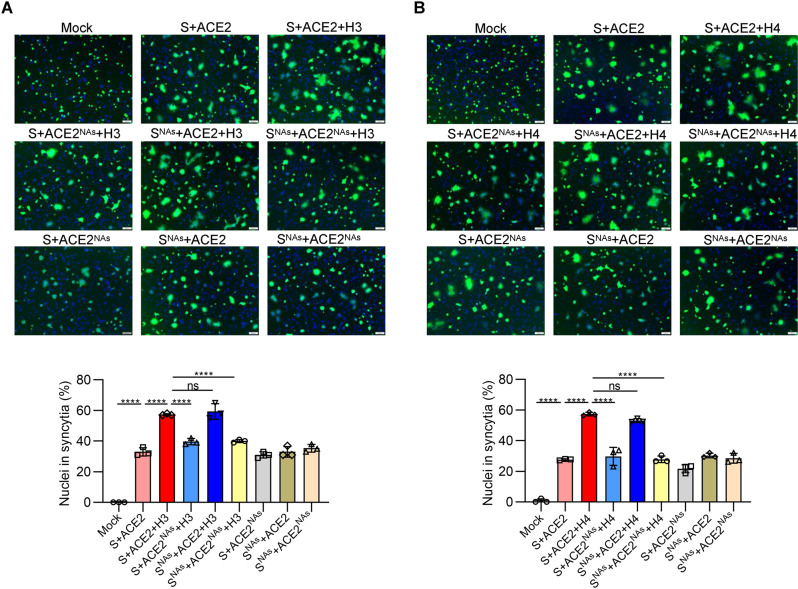
Effect of histone H3 or H4 and sialic acid on cell–cell fusion. Representative images (top) and quantitative analysis (bottom) of syncytia in the cell–cell fusion mediated by SARS-CoV-2 S protein in the presence or absence of histone H3 (**A**) or H4 (**B**). The effector cells or target cells were pretreated with neuraminidase for 12 h, as described in the “Methods”. NAs neuraminidase. Scale bars represent 100 μm. One-way ANOVA followed by Tukey’s multiple comparison post hoc test was conducted. All error bars represent the SEM. *P < 0.05; **P < 0.01; ***P < 0.001; ****P < 0.0001; ns not significant

SARS-CoV-2 utilizes plasma membrane fusion as a key event to enter host cells [3]; thus, we determined the effects of histones and sialic acid on the membrane fusion process. It is not surprising to find that the cell–cell fusion mediated by SARS-CoV-2 S protein was enhanced by the addition of either histone H3 or H4 (Fig. 4A, B). The enhanced cell–cell fusion mediated by histones was inhibited by pretreatment of 293T cells expressing ACE2 with NA but not by the same pretreatment of 293T cells expressing SARS-CoV-2 S protein. In addition, NA had no effect on cell–cell fusion without histones (Fig. 4A, B). These findings suggest that histones may also enhance cell–cell fusion by bridging the SARS-CoV-2 S protein with sialic acid on host cells.

### The effect of histones on SARS-CoV-2 infectivity in a mouse model

**Fig. 5:**
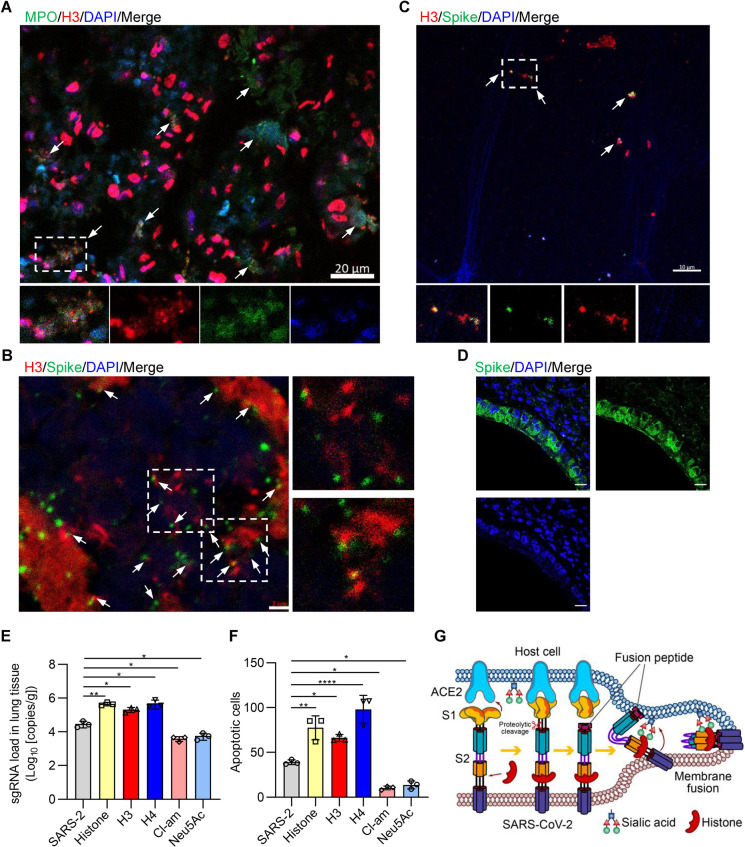
The effect of histones on the infectivity of SARS-CoV-2 in a mouse model. **A** Confocal immunofluorescence images of NETosis of neutrophils with the markers MPO (green), histone H3 (red), and DAPI (blue) in infected mouse lung tissues. The arrow indicates MPO-positive neutrophils undergoing NETosis. The inset in the merged image is the magnified image of a representative cell (arrow). **B** The representative confocal immunofluorescence images show conjugates (arrow) formed by spike protein (green) and histone H3 (red). The inset in the merged image is the magnified image of the conjugates (arrow). **C** The confocal immunofluorescence images show conjugates formed by spike protein (green) and histone H3 (red) released by neutrophils in vitro. DAPI served as a nuclear DNA counterstain (blue). The inset in the merged image is the magnified image of the conjugates (arrow). **D** The representative images showed S protein-positive (green) staining that appeared within the cytoplasm of the cells lining the bronchioles. **E** RT-qPCR was used to measure sgRNA in lung tissues in each group. Data are individual values and geometric means. **F** Quantification of the apoptotic (TUNEL-positive) cells in lung tissues in each group. **G** Graphic illustration showing that the S1 receptor binding domain of the SARS-CoV-2 S protein binds to the ACE2 receptor on the host cell, followed by activation of the S protein by proteolytic cleavage, detachment of the S1 domain from the viral surface, insertion of the exposed hydrophobic fusion peptide of the S2 domain into the host cell membrane, formation of a six-helix bundle (6-HB) fusion core by the heptad repeat 1 (HR1) and 2 (HR2) domains of subunit 2 and fusion of the viral and cellular membranes. In this process, the histones released by NETosis bridge the SARS-CoV-2 S2 subunit and sialic acid on the cells to promote membrane fusion, thereby facilitating SARS-CoV-2 cell entry and infectivity. Scale bars represent 20 μm in (**A**), 2 μm in (**B**), 10 μm in (**C**) and 10 μm in (**D**). Student’s *t* test was performed for (**E**), and SARS-2 was used as a control. One-way ANOVA followed by Tukey’s multiple comparison post hoc test was conducted in (**F**). All error bars represent the SEM. *P < 0.05; **P < 0.01; ***P < 0.001; ****P < 0.0001; ns, not significant

We also investigated whether the enhanced infectivity of SARS-CoV-2 mediated by histones occurs in a mouse model and its significance in the development of potential therapeutics for SARS-CoV-2 infection. We have reported that a mouse model with pathological changes characteristic of SARS-CoV-2-induced acute respiratory distress syndrome was established by intratracheal instillation of SARS-CoV-2, with predominant neutrophil infiltration in lung tissues as early as 6 h after the inoculation of the virus [33]. It is an ideal model to investigate the role of neutrophils in response to SARS-CoV-2. In this study, we found apparent NETosis of neutrophils in the infected lung tissues (Fig. 5A). Namely, MPO-positive neutrophils showed the morphological characteristics of NETosis, with the nuclear envelope disintegrated, the plasma membrane ruptured, and decondensed nuclear chromatin structures mixed with H3 or H4 histones and MPO (Fig. 5A). Histone debris or nuclear fragments released from neutrophils undergoing NETosis were frequently found in the extracellular space in the infected lung tissues (Fig. 5A, B). It was easy to find that histone H3- or H4-positive debris colocalized with S protein-positive tiny particle-like structures, often forming conjugates, as shown by confocal microscopy (Fig. 5B). Similarly, we found that SARS-CoV-2 triggered neutrophil NETosis in vitro with S protein- and histone H3-positive conjugates (Fig. 5C and Fig. S7). These findings suggest that the binding of histones with the S protein of SARS-CoV-2 or their conjugates on the cell surfaces may occur in lung tissues and may be present at the early stage of infection, namely, before the cell entry of SARS-CoV-2. However, after the viruses entered and replicated within the cells, S protein-positive staining appeared within the cytoplasm of the cells lining the bronchioles or the alveolar spaces (Fig. 5D). Moreover, we treated mice infected with SARS-CoV-2 with inhibitors of NETosis, free sialic acid, total histone, histone H3 and histone H4, investigated their effects on the infectivity of SARS-CoV-2, and recorded the apoptotic cells in the lung tissues. Treatment with Cl-amidine or free sialic acid significantly reduced the level of sgRNA copies (Fig. 5E), which is indicative of viral replication and the number of apoptotic cells (Fig. 5F). In contrast, treatment with total histone, histone H3 or histone H4 induced increased levels of sgRNA copies (Fig. 5E) and an increased number of apoptotic cells (Fig. 5F). These findings indicated that the enhanced infectivity of SARS-CoV-2 mediated by histones also occurs in a mouse model, and the blocking of histone H3 or histone H4 and its binding sites may inhibit the infectivity of SARS-CoV-2 and reduce cell death in lung tissues.

## Discussion

NETs are known to capture and eliminate viruses, such as influenza viruses, HIV, and RSV, thereby preventing viral spread [11–13]. However, in this study, SARS-CoV-2 was shown to hijack histones from neutrophil NETosis to promote its host cell attachment and entry process. The enhanced virus infectivity may result from the positively charged histones (especially histone H3 and H4), which act as a key mediator bridging the negatively charged spike subunit 2 protein and sialic acid on the host cells. This demonstration is supported by our findings in the present study as follows. Enhanced infectivity of SARS-CoV-2 induced by NETosis or histones was found using the live SARS-CoV-2 system, two pseudovirus systems, and a mouse model with live virus. Histone H3 or H4 selectively bound to S2, as observed by biochemical binding assay, surface plasmon resonance (Biacore), the calculation of the binding energy, and the construction of the mutant S protein by replacing four acidic amino acids. Sialic acid on the host cell surface is the key molecule to which histones bridge S2, as evidenced by the cell binding blocking assay using NA or sialic acid. Moreover, histones enhance cell–cell fusion by bridging the SARS-CoV-2 S protein and sialic acid on host cells. Finally, in a SARS-CoV-2-induced acute respiratory distress syndrome mouse model, treatment with inhibitors of NETosis, histone H3 or H4, or sialic acid notably affected the levels of sgRNA copies and the number of apoptotic cells in vivo.

Many studies have reported that viruses can induce NETosis of neutrophils in vitro, followed by NET capture and inactivation [11–13]. It is commonly believed that citrullination of histones by PAD4 is required for the formation of NETs, which results in a reduction in the positive charge of the modified proteins in the NET by the conversion of arginine residues to peptidylcitrulline [18]. However, recent studies demonstrated that elevated histones, instead of citrullinated histones, were present during the process of NETosis [19–22]. Moreover, extracellular histones have been widely used for research on NET-mediated cytotoxicity or antiviral effects in many previous studies [17, 25, 27]. Thus, there is reason to accept that the release of free histones with a positive charge could occur in NETs, and it is reasonable to use recombinant histones in our research. As the most abundant proteins in NETs, histones are enriched in highly positively charged amino acids and can attach to negatively charged viral envelopes, thereby eliminating viruses. For example, extracellular histones can reduce viral transcription and inactivate viruses [12, 17, 34]. In this study, we examined whether NETs or neutrophil NETosis have the same impact on the infectivity of SARS-CoV-2. Contrary to our expectation, histones released from NETs could enhance the infectivity of SARS-CoV-2. Importantly, the enhanced infectivity of SARS-CoV-2 can be attributed to histones selectively binding to the negatively charged amino acids in the acidic C-terminal domain of S2. Some studies have revealed that S1 of SARS-CoV-2 can bind to other receptors or coreceptors, such as neuropilin-1, heparan sulfate, HDL-scavenger receptor B type 1, tyrosine-protein kinase receptor UFO (AXL) and CD147 [5–10]. However. to the best of our knowledge, no study has reported that the receptor or coreceptor can bind to S2 to facilitate SARS-CoV-2 cell entry.

Sialic acid is an acidic monosaccharide and is present in all living cells as glycoconjugates to attach to various glycoproteins and glycolipids, which are essential for maintaining the negative charge of the cell surface [32]. In our study, we also identified sialic acid as a novel binding site on the host cell surface that bridges S2 with the host surface via histones. Heparan sulfate, a linear polysaccharide with a highly negative charge, is located on a small set of membranes or extracellular matrix proteoglycans [7]. A recent study reported that the RBD of the SARS-CoV-2 S protein could bind to heparin sulfate through a docking site composed of positively charged amino acid residues [7]. Consistent with this study, treatment with heparin lyases (HSase) reduced the infectivity of pseudovirus. However, removing heparin on the cell surface did not reduce the effect of histone H3 or H4 on the infectivity of pseudovirus, which indicated that the enhanced infectivity mediated by histones occurs through a different system from a heparin sulfate-binding site adjacent to the ACE2-binding site of S1.

Finally, we briefly summarize the possible role of our new findings in viral and cellular membrane fusion and infection. As illustrated in Fig. 5G, SARS-CoV-2 first binds to the host cell surface ACE2 receptor via the RBD in S1. Viral attachment and host protease cleavage facilitate conformational changes in the S protein and the insertion of the fusion peptide of the S protein into the target membrane. Subsequently, the heptad repeat 1 (HR1) and 2 (HR2) domains in S2 interact with each other to form a stable six-helix (6-HB) bundle fusion core and eventually accelerate the viral and cellular membrane fusion process [3, 7, 35, 36]. Our new findings may indicate additional bridging by histones between spike subunit 2 protein and sialic acid on the host cells, bringing viral and cellular membranes into proximity for fusion and infection. In summary, histones and sialic acid may be important factors in host cells that regulate the infectivity of SARS-CoV-2 via S2, and this finding may be of importance to explore the pathogenesis of COVID-19 and a possible strategy to develop new therapies.

## Materials and methods

### Preparation of pseudovirus

Pseudovirus with spike proteins of SARS-CoV-2 was generated as described previously [31]. Briefly, 293T cells were transfected with a plasmid encoding codon-optimized SARS-CoV-2 spike protein with a C-terminal 18 aa truncation, a lentiviral vector carrying either EGFP (for FACS assay) or Luc2 (for luciferase assay) reporter, and a gag/pol expression plasmid (Addgene, 12260) using polyethyleninime (Polyscience). Six hours post transfection, the medium was replaced with new complete culture medium. Forty-eight hours post infection, the culture supernatants containing pseudovirus were harvested, filtered through a 0.45 μM pore-size filter (Millipore, SLHP033RB), subjected to ultracentrifugation, and stored at −80 °C prior to infection assays. EGFP or luciferase expression in infected 293 T/ACE2 cells was determined by fluorescence microscopy, flow cytometry or a multimode microplate reader (PerkinElmer).

### Infection by live SARS-CoV-2 or pseudovirus

Vero E6 cells (5 × 10^4^) were plated in 96-well tissue culture plates and grown overnight. Neutrophils (1 × 10^5^) obtained from healthy humans were incubated with SARS-CoV-2 (MOI = 0.01 or 0.05) with or without PMA (Sigma-Aldrich) (10–100 nM) at 37 °C for 3 h. Then, the supernatant was collected and added to Vero E6 cells. After 48 h, the cytopathogenic effects were recorded using a microscope, and the percentages of cells with cytopathogenic effects were calculated as reported previously [31]. To inhibit NET release, neutrophils were pretreated with Cl-amidine (Selleck, S8141) (50–200 μM) 1 h before incubation with SARS-CoV-2.

Neutrophils were incubated with culture supernatant containing luciferase- or EGFP-expressing SARS-CoV-2 pseudovirus or with or without PMA (10–100 nM) at 37 °C for 3 h. To determine the infectivity of the pseudovirus in the supernatant, the mixed supernatant was added to ACE2-expressing HEK-293T (293T/ACE2) cells in serum-free DMEM. The medium was changed to complete DMEM after 6 h, followed by incubation for 48 h to express the reporter gene. The efficiency of viral entry was determined with a firefly luciferase assay, fluorescence microscopy, flow cytometry, or a multimode microplate reader (PerkinElmer).

To test the effect of histones on the infectivity of pseudovirus, the pseudovirus was preincubated with total histone (1–5 μg/ml), H2A (Huabio) (0.5–25 μg/ml), H2B (Huabio) (0.5–15 μg/ml), H3 (Huabio) (0.5–5 μg/ml) or H4 (Huabio) (0.5–5 μg/ml), keeping the indicated final concentrations in serum-free DMEM containing the indicated inhibitors or antibodies at 37 °C for 3 h. Then, the mixtures were added to 293 T/ACE2 cells or ACE2-negative cells, and the efficiency of viral entry was determined through a firefly luciferase assay after 48 h. For the EGFP-expressing pseudovirus entry assay, the number of EGFP-positive cells was determined with fluorescence microscopy and flow cytometry. To block the effects of histones H3 and H4 on the infectivity of pseudovirus, H3 or H4 was preincubated with H3 Ab (Huabio) (10 μg/ml) or H4 Ab (Huabio) (10 μg/ml) and Neu5Ac (free sialic acids) (Selleck, S4792) (10 μg/ml) or PSA (Sigma-Aldrich, C5762) (10 μg/ml), followed by incubation with pseudovirus. Mouse IgG (Abcam, ab18447) (same dose as H3 Ab) and rabbit IgG (Huabio) (same dose as H4 Ab) were used as controls in the antibody blocking assay. In some experiments, 293 T/ACE2 cells were pretreated with NA (Sigma-Aldrich, N2876) (0.1 U/ml) or HSase (heparin lyase) mix (Sigma-Aldrich, H2519, H6512, H8891) (2.5 mU/ml HSase I, 2.5 mU/ml HSase II, and 5 mU/ml HSase III).

To investigate the effect of histones on the infectivity of live SARS-CoV-2, SARS-CoV-2 (MOI = 0.01 or 0.05) was incubated with total histone (Sigma-Aldrich), histone H3 (Huabio), or histone H4 (Huabio) in serum-free DMEM at 37 °C for 3 h in a similar way to the pseudovirus described above. Then, the mixture was added to Vero E6 cells (5 × 10^4^). After 6 h, complete DMEM was added to maintain cell survival. The cytopathogenic effects were recorded and calculated after 48 h.

### Binding of the spike protein to histones

The flat-bottom 96-well high binding plates (NUNC-MaxiSorp, Thermo Fisher Scientific) were coated with 1 μg/ml of histone H3 or H4 dissolved in 200 μl of carbonate coating buffer (50 mM, pH 9.6) per well at 4 °C overnight. The plates were washed three times with PBS containing 0.1% Tween-20 (PBST). The biotin-tagged spike (Sino Biological, 40589-V08B1-B), S1 subunit (Sino Biological, 40591-V08H-B), S2 subunit (Sino Biological, 40590-V08B-B), RBD (Sino Biological, 40592-V08B-B) or ACE2 proteins (Sino Biological, 10108-H27B-B) at concentrations of 0, 1, 5, 10, 25, 50, or 100 nM were added to each well and incubated at room temperature for 30 min. Each well was washed three times with PBST. Streptavidin-conjugated horseradish peroxidase (HRP) was added to each well and incubated for 30 min. Then, the wells were washed five times with 200 μl of PBST and developed with TMB substrate. The reaction was quenched by the addition of 50 μl of 1.0 M H_2_SO_4_ solution. The absorbance was measured at 450 nm.

### Surface plasmon resonance analysis

SPR-based measurements were performed by Biacore (8 K) as previously described [37]. Briefly, the histone H3 or H4 proteins were immobilized on a CM5 sensorchip (Cytiva) to a level of 25 response units (RUs) using Biacore (8 K). For affinity analysis, the spike protein (S1 + S2), S1 subunit or S2 subunit were dissolved in HBS-EP + running buffer at concentrations of 78.125, 156.25, 312.5, 625, and 1250 nM and were run across the chip. Each sample that was bound to the antigen surface was dissociated by HBS-EP+ buffer for 120 s at a flow rate of 30 μl/min. Regeneration of sensor chips was performed for 60 s using regeneration buffer (glycine pH 1.5). The dissociation constant (*K*_D_) was determined and recorded by Biacore (Cytiva).

### Molecular modeling of SARS-CoV-2 S protein and histone

We used the rigid-body protein–protein docking algorithm ZDOCK [38] to systematically search the rotational and translational space of SARS-CoV-2 S protein and histone. The trimeric S protein of SARS-CoV-2 (PDB ID 6VSB) [39] was used as the receptor protein, and the histone (PDB ID 6HKT) [40] was used as the ligand protein. We then refined and evaluated the interaction energies of docked conformations using the ZRANK algorithm [41], which uses a CHARMm-based scoring function to calculate the energy of docked poses. The docked poses were filtered based on the ZRANK score. Two thousand structures were retained and clustered using the pairwise root mean square error (RMSD) as the distance measure. Finally, the docked poses were discarded if clashing between the histones and the S protein N-glycans was detected. An electrostatic potential map of the C-terminus of the SARS-CoV-2 S protein was generated from a crystal structure (PDB ID 6VSB) and visualized using Discovery Studio 3.1. For the calculation of the binding energy, molecular dynamics (MD) simulations were performed using NMAD [42]. The structures of proteins extracted from the thermodynamic equilibration trajectories were used to calculate the interaction binding energy and the energy contributions of the critical residues. The initial structures were prepared based on cryo-EM or X-ray crystal structures: SARS-CoV-2 S protein (PDB ID 6VSB), histone H3 and H4 (PDB ID 6HKT), and sialic acid (PDB ID 2CWG). The unresolved residues were constructed using Modeller 9.23 [43]. For the S protein mutant simulations, D1139P, E1144K, E1150S, and D1153S were constructed from cryo-EM structure 6VSB using Modeller. To allow sufficient optimization of the interaction models of histones and sialic acid, the sialic acid molecules were randomly placed inside the simulated systems as the starting models of MD simulations.

All-atom MD simulations were performed in the *NPT* ensemble. All simulated models were immersed in a TIP3P water box with a 15 angstrom edge length. Constant pressure (*P* = 1 bar) and temperature (*T* = 300 K) were maintained using the Langevin piston coupling algorithm. The charmm 36 force field [44] was employed for the protein. The charmm CGenFF force field [45] was used for sialic acid. The charge states of protein ionizable groups were normalized to pH 7.0. Sodium (Na^+^) and chloride (Cl^–^) counterions were added to ensure global charge neutrality at a physiological concentration of 0.15 M using VMD. The SHAKE algorithm was used to constrain the lengths of all chemical bonds involving hydrogen atoms. The integration time step of the simulations was set to 2.0 fs. Nonbonded van der Waals interactions were treated by using a switching function at 10 Å. Long-range electrostatic forces were handled by using the particle mesh Ewald algorithm, which is an efficient method for periodic boundary conditions. The systems were minimized using the steepest-descents algorithm and heated from 50 K to 300 K with fixed protein backbone atoms. The systems were then submitted to NAMD for 10 ns NPT equilibrations before all-atom MD productions with the whole system relaxed.

### Spike mutagenesis

There are three acidic submotifs (1139–1153 aa, 1163–1168 aa, and 1184–1199 aa) in the acidic domain of S2. The potential histone-binding site calculated by the docking program was located in the first submotif. To verify whether this acidic submotif is the histone-binding site, we generated pseudoviruses carrying mutations (mut-1 and mut-2) in these submotifs by replacing the acidic amino acids with neutral or base residues. To prevent dramatic destruction of the native S protein structure, multiple sequence alignment of the coronavirus family members was performed using Clustal Omega to check the conservation level of mutation sites and to guide the amino acid substitution. Mut-1 covers the negatively charged amino acids and contains four point mutations (D1139P, E1144k, E1150S, and D1153S). Mut-2covers the other negatively charged amino acids, including seven point mutations (D1163A, D1165N, D1168S, D1184N, E1188Q, E1195N, and D1199N). Spike mutagenesis was performed with two-fragment PCR and Gibson-Assembly approach by using Gibson Assembly Master Mix (NEB, E2611) according to the instructions.

### Binding of histone H3, H4, or S2 subunit proteins to the cell surface

Cell surface binding of H3 and H4 was detected by flow cytometry. Biotin-tagged histone H3 (10 ng/ml) or H4 (35 ng/ml) protein was added to Calu-3 cells in PBS buffer. After incubation at room temperature for 30 min, the cells were washed three times with PBS and then stained with a PE-conjugated antibody (Biolegend, MX2013931) (PE antibiotin) at 4 °C for 30 min. To block the binding of H3 or H4 to cells, H3 or H4 proteins were preincubated with Neu5Ac (10 μg/ml) or pretreated with NAs (0.1 U/ml) for 24 h.

For the SARS-CoV-2 S2 subunit protein and cell surface binding assays, the biotin-tagged S2 subunit (5 nM) was preincubated with histone H3 or H4 at 37 °C for 20 min. The cells were then incubated with the mixture for 30 min. The cells were washed and stained with PE antibiotin antibody. Binding was detected by a flow cytometer (ACEA Biosciences), and the results were analyzed with FlowJo V10 software. In some experiments, a portion of the cells was pretreated with NAs (0.1 U/ml) in DMEM for 24 h at 37 °C.

### Cell–cell fusion assay

The establishment and detection of the SARS-CoV-2 S-mediated cell–cell fusion assays were described previously [3, 46]. Briefly, HEK-293T cells cotransfected with a plasmid encoding EGFP (pEGFP-C1) and a vector encoding the SARS-CoV-2 S glycoprotein with a C-terminal 18-aa truncation (293T/SARS-CoV-2 S/EGFP) were used as effector cells. 293T cells expressing human ACE2 receptors on the membrane surface (293T/ACE2) were utilized as target cells. Before the cell–cell fusion assays, the effector cells and target cells were treated with NA for 12 h. Effector cells and targeted cells were cocultured at a ratio of approximately one SARS-CoV-2 S protein-expressing cell to one ACE2 receptor-expressing cell in the absence or presence of histone H3 (1 μg/ml) or histone H4 (2 μg/ml) at the final concentration as indicated. After further coculture at 37 °C for 4 h, the cells were stained with Hoechst (Beyotime Biotechnology, Hoechst 33342), and syncytium formation between 293T/SARS-CoV-2/EGFP and 293T/ACE2 cells was observed under an inverted fluorescence microscope. 293T/EGFP cells were used as the negative control. Three fields were randomly selected in each well to count the number of fused and unfused cells.

### Mice and experimental protocol

Transgenic hACE2 mice (8–10 weeks) on a C57BL/6 background were provided by the National Institutes for Food and Drug Control. The mouse model with the pathological changes of SARS-CoV-2-induced acute respiratory distress syndrome was established by intratracheal instillation, as previously described [33]. Briefly, the mice were anesthetized with 5% isoflurane, a small superficial incision was made in the midline of their necks to expose the trachea, and 4 × 10^5^ PFU of SARS-CoV-2 in 60 μl of PBS was intratracheally instilled with a 29-gauge insulin syringe (Becton, Dickinson and Company, USA). In the total histone, histone H3 and histone H4 groups, 4 × 10^5^ PFU of SARS-CoV-2 in 40 μl PBS was mixed with total histone, histone H3 or histone H4 at the indicated dose, and the final volume was kept at 60 μl. After incubation at 37 °C for 3 h, the mixtures were intratracheally instilled. After instillation, the overlying skin was closed with wound clips, and the animals were placed on a heating pad in their cage until they recovered from anesthesia. In the Cl-amidine- and Neu5Ac-treated groups, the mice were intraperitoneally (i.p.) injected with a total of 200 μl (50 mg/kg) of Cl-amidine or 200 μl (20 mg/kg) of Neu5Ac immediately after the SARS-CoV-2 challenge and were continuously injected once per day for five consecutive days.

All mice were euthanized by isoflurane overdose 5 days post infection for serum collection and tissue processing. Lung tissues were collected for immunofluorescence (IF) staining and for RT-qPCR assays. All procedures associated with animal study were reviewed and approved by the Institutional Animal Care and Use Committee of Institute of Medical Biology, Chinese Academy of Medical Sciences, and were performed in the ABSL-4 facility of Kunming National High-level Biosafety Primate Research Center.

### RT-qPCR assay

SARS-CoV-2 E gene subgenomic mRNA, indicative of virus replication, was assessed by RT-qPCR as previously described [31, 47], using the following primer and probe sequences: forward, 5′-CGATCTCTTGTAGATCTGTTCTC-3′; reverse, 5′-ATATTGCAGCAGTACGCACACA-3′; probe, 5′-FAM-CGAAGCGCAGTAAGGATGGCTAGTGT-Quencher-3′.

### Immunofluorescence (IF) staining

Lung tissues were harvested and used for IF staining [48]. Briefly, after incubation with blocking buffer (5% normal goat serum) for 10 min at room temperature, the slides were incubated with primary antibodies overnight at 4 °C. After five washes, the sections were incubated with secondary antibody at 37 °C for 1 h. In the in vitro experiment, neutrophils were allowed to attach to the coverslips and incubated for 4 h at 37 °C for immunostaining. The primary antibodies used for IF analysis included rabbit anti-MPO (Abcam, ab9535), rabbit anti-SARS-CoV-2 Spike (Sino Biological, 40589-T62), and recombinant anti-histone H3 (methyl K37) antibodies (Abcam, ab215728). Secondary antibodies included goat anti-rabbit IgG H&L (Alexa Fluor^®^ 488) (Abcam) and goat anti-rat IgG H&L preadsorbed (Alexa Fluor^®^ 647) (Abcam, ab150167).

### TUNEL assay

The TUNEL assay was performed according to the instructions of the DeadEnd^TM^ Fluorometric TUNEL System (Promega, USA). The results were observed by fluorescence microscopy (Leica, Germany). The number of positive cells was calculated from the observation of three random fields.

### Statistical analysis

The statistical analyses were carried out using Prism software (GraphPad Prism 8.0). Comparisons between two groups were performed using unpaired Student’s *t* tests. Comparisons among multiple groups were performed using one-way ANOVA followed by Tukey’s multiple comparison post hoc test. *P* < 0.05 was considered significant (significance is denoted as follows: ns no significance; *P < 0.05; **P < 0.01; ***P < 0.001; ****P < 0.0001).

## Supplementary information

Supplemental Information

## Data Availability

The study did not generate any unique datasets or codes.
