# Supplementary material for: Histones released by NETosis enhance the infectivity of SARS-CoV-2 by bridging the spike protein subunit 2 and sialic acid on host cells
Source: Cell Mol Immunol. 2022 Mar 10;19(5):577–87. doi: 10.1038/s41423-022-00845-6 (PMC8907557; doi:10.1038/s41423-022-00845-6)
Supplement: Supplementary file 1 — Supplemental Information [file 41423_2022_845_MOESM1_ESM.docx]

**Supplemental Information**

Figures S1-S6

**Histones released by NETosis enhance the infectivity of SARS-CoV-2 by bridging spike subunit 2 protein and** **sialic acid on the host cells**

**
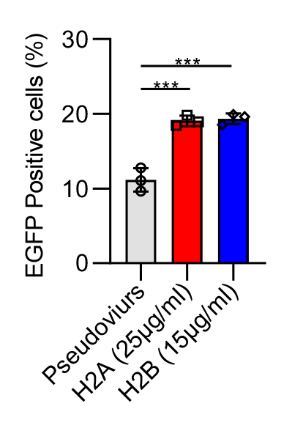
**

**Fig S1. The effect of histone H2A or H2B on the infectivity of EGFP-expressing pseudovirus.**

The supernatant containing EGFP-expressing pseudovirus were incubated with histone H2A (25 μg/ml) or H2B (15 μg/ml) at 37℃ for 3 hours. The mixture was then added to 293T/ACE2 cells. The number of EGFP positive cells were determined by a flow cytometry and observed with a fluorescent microscopy. One-way ANOVA analysis followed by Tukey’s multiple comparison post hoc test was conducted. All error bars represented SEM about the mean. **P*< 0.05; ***P* < 0.01; ****P*< 0.001; *****P* <0.0001.


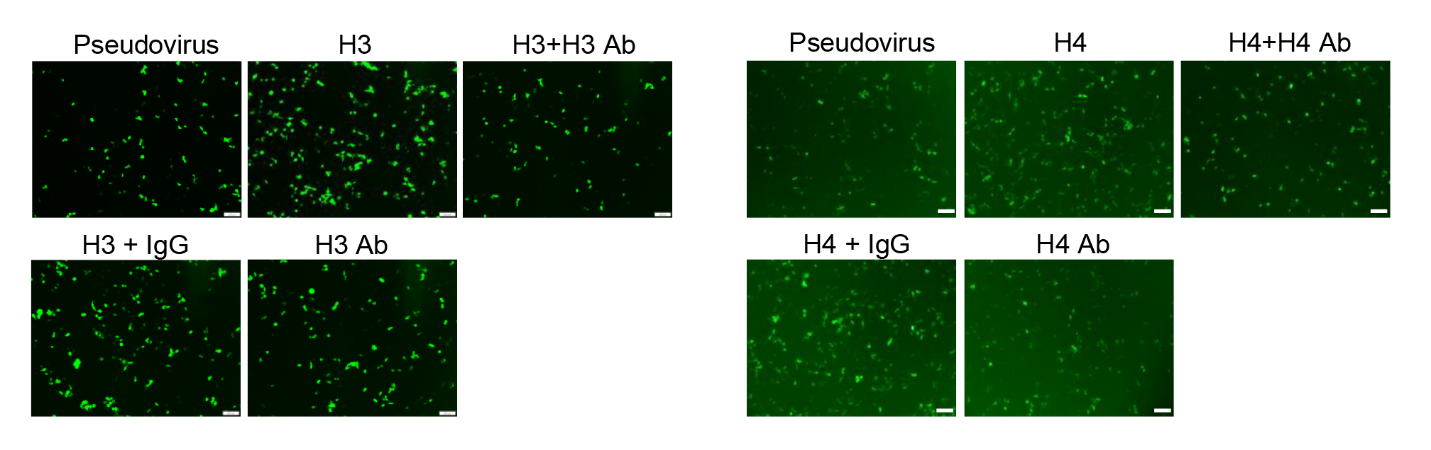


**Fig S2. The enhanced infectivity mediated by histone H3 or H4 was blocked by the antibody against with H3 or H4.**

The fluorescent images showed that the 293T/ACE2 cells were infected with the supernatant-containing pseudovirus plus histone H3 (left) or H4 (right) pre-treated with the antibody against histone H3 or H4, or with control IgG. Scale bars represent 100 μm.


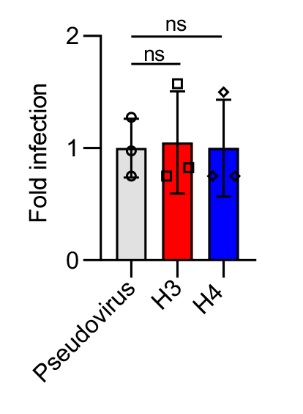


**Fig S3. No enhanced infectivity mediated by histones in ACE2-negative HEK-293T cells.**

The infectivity of pseudoviurs pre-incubated with histone H3 or H4 was determined in non-transfected ACE2-negative HEK-293T cells. One-way ANOVA analysis followed by Tukey’s multiple comparison post hoc test was conducted. All error bars represented SEM about the mean. **P*< 0.05; ***P* < 0.01; ****P*< 0.001; *****P* <0.0001; ns, not significant.


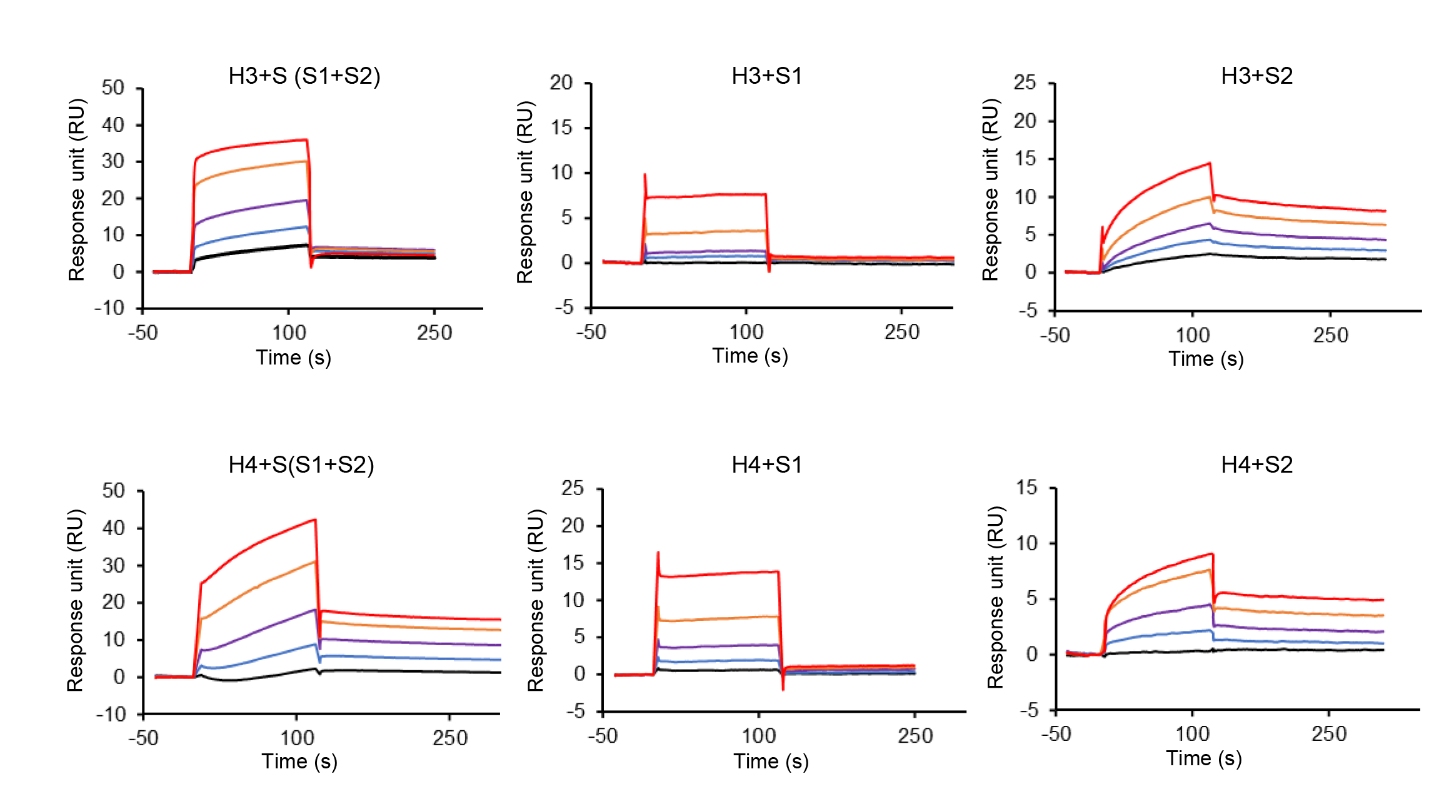


**Fig S4. The real time binding profile between spike protein (S1+S2), subunit 1, subunit 2 protein and histone H3 or histone H4.**

The surface plasmon resonance (Biacore) performed as described in Methods, histone H3 and H4 were immobilized to a CM5 sensorchip. Spike protein (S1+S2), subunit 1 or subunit 2 proteins were dissolved at the concentration of 78.125, 156.25, 312.5, 625, 1250 nM, and the dissociation constant (K_D_) was determined by Biacore.


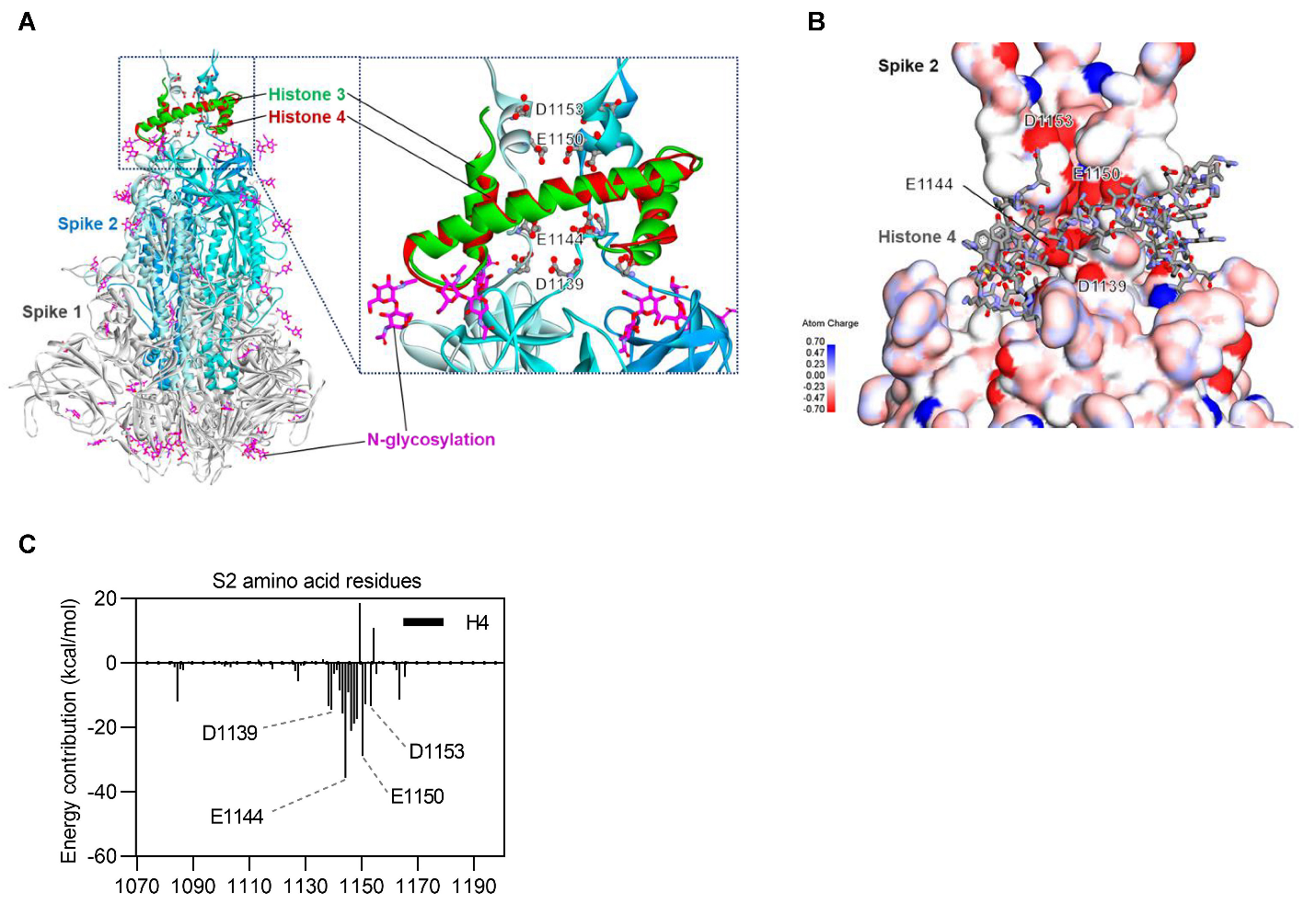


**Fig S5. Molecular simulation to investigate the interaction between histones and SARS-CoV-2 spike protein.**

**A** A molecular model of SARS-CoV-2 S protein trimer (PDB ID 6VSB) in complex with histones H3 and H4. Proteins are represented using standard ribbon format. subunit 1 is shown in gray, subunit 2 protein in blue, histone H3 in green, and histone H4 in red. The N-glycosylation are represented using magenta stick (left panel). A set of negatively charged residues lies in the C terminal of subunit 2 (right panel).

**B** Electrostatic surface rendering of the C terminal of subunit 2 protein (PDB ID 6VSB) in complex with histone H4 (shown in sticks). Blue and red surfaces indicate electropositive and electronegative surfaces, respectively. The important binding residues are labeled.

**C** Calculated energy contributions of each amino acid residue of the subunit 2 protein that can interact with H4.


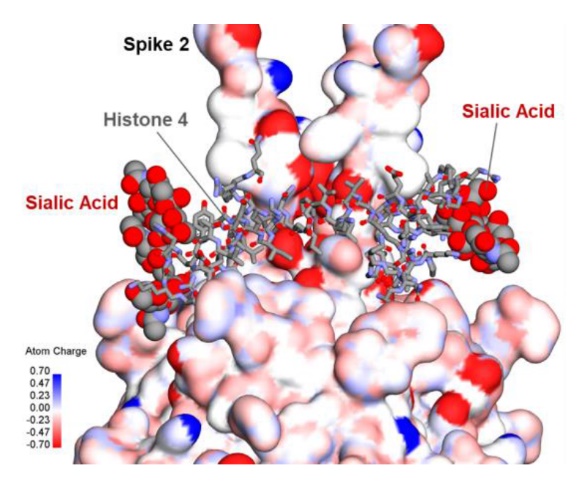


**Fig S6. The binding of histone H4 with sialic acids.**

A molecular model of the histone H4 forming multivalent interactions with both SARS-CoV-2 spike 2 protein and sialic acids. Sialic acids are represented using standard sphere model.


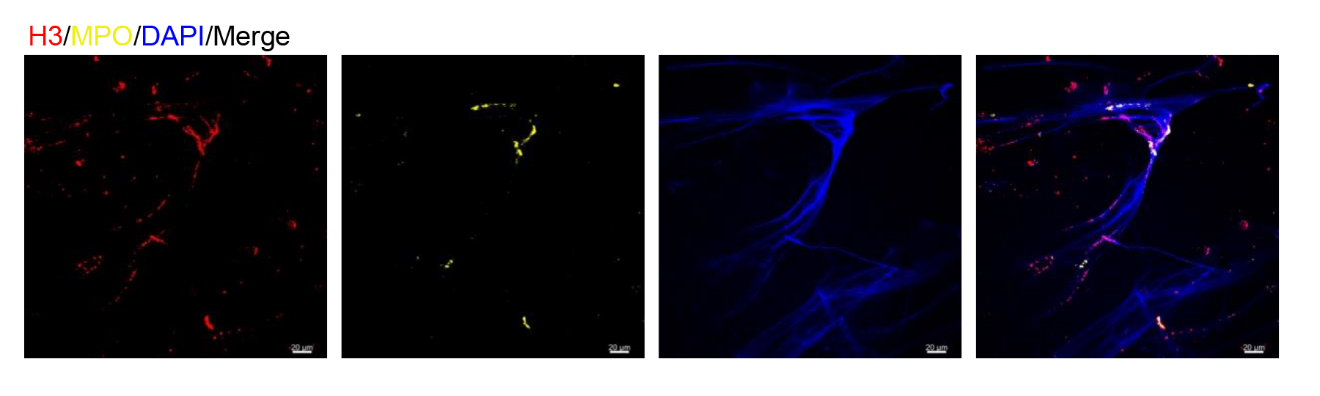


**Fig S7. SARS-CoV-2 triggered neutrophil NETosis *in vitro*.**

The representative images of NETosis of human neutrophil triggered by SARS-CoV-2 *in vitro*. Histone H3 (red), MPO (yellow) and DAPI (blue). Scale bars represent 20 μm.
